# Comprehensive Analysis of Sorghum CNGC Genes Reveals Their Potential Roles in Abiotic Stress Responses

**DOI:** 10.3390/genes16121405

**Published:** 2025-11-25

**Authors:** Yu Luo, Wenda Jiao, Kun Huang, Xiang Li, Jiaqi Li, Minli Wang, Ruidong Zhang, Xiong Cao

**Affiliations:** 1 College of Agronomy, Shanxi Agricultural University, Taigu, Jinzhong 030801, China; 2 Institute of Industrial Crops, Shanxi Agricultural University, Taiyuan 030031, China

**Keywords:** sorghum, CNGC gene family, abiotic stresses

## Abstract

**Background/Objectives**: Cyclic nucleotide-gated channel (CNGC) genes play crucial roles in plant growth, development, and stress responses, yet their functions in sorghum remain poorly understood. **Methods**: This study systematically analyzed sorghum CNGC genes through genome-wide identification, encompassing chromosomal mapping, phylogenetic relationships, gene structure, cis-acting elements, miRNA regulation, and GO/KEGG annotation. **Results**: A total of 23 sorghum CNGC genes were identified and classified into five subclasses (I–IV-b), exhibiting high evolutionary conservation with rice and maize. Promoter and miRNA analyses revealed multi-level regulation involving light, hormones (ABA, JA), and stress response elements. Several SbCNGC genes were predicted to be regulated by multiple miRNAs. Expression profiling and qRT-PCR validation indicated that most SbCNGC genes responded to both high-temperature and low-temperature stress. Expression analysis revealed tissue specificity and stress-induced transcriptional responses. Notably, SbCNGC1 remains consistently upregulated under both cold and heat stress, suggesting a potential key role in Ca^2+^-mediated signaling. **Conclusions**: This study systematically characterizes SbCNGC genes for the first time, reveals their potential role in abiotic stress tolerance, and provides a valuable resource for sorghum functional genomics and molecular breeding.

## 1. Introduction

Ion channels are important transporter proteins in plant cell membranes that directly affect mineral nutrient uptake and homeostasis by regulating ions’ transport across membranes [1]. These mineral elements (e.g., K^+^, Ca^2+^, Mg^2+^, NO_3_^−^, etc.) are essential for plant growth and development. They are involved in physiological and biochemical processes, including the regulation of enzyme activity, maintenance of osmotic pressure, photosynthesis, and signal transduction [2]. For example, calcium ions (Ca^2+^) act as second messengers, regulating several signaling pathways involved in growth and development, as well as responding to environmental stimuli in biotic and abiotic stress [3]. Therefore, functional deficiencies or abnormalities in ion channels often lead to imbalances in plant mineral nutrition, which in turn affect growth, yield, and stress tolerance. The maintenance of intracellular Ca^2+^ homeostasis in plants depends on the synergistic regulation of multiple transporters. In addition to cyclic nucleotide-gated channels (CNGCs), glutamate-like receptors trigger Ca^2+^ inward flow by sensing extracellular signals [4], whereas hyperosmotic-induced Ca^2+^ increase channels (OSCAs) mediate Ca^2+^ signaling under osmotic stress [5]. Like these transporters, Cyclic Nucleotide-Gated Channels (CNGCs) participate in Ca^2+^ dynamic homeostasis through non-selective cation transport [6].

CNGCs are an important class of non-selective cation channels, widely found in plants and animals, that are involved in key biological processes such as plant growth and development, biotic and abiotic stress response, and signaling by mediating the transmembrane transport of ions such as Ca^2+^, K^+^, etc. [7]. These channels consist of a heterotetrameric complex of 2–3 different subunits, and their activity is subject to multiple fine regulations: activation by direct binding of cyclic nucleotides (cAMP/cGMP), negative feedback regulation by Ca^2+^/calmodulin complexes, and dynamic control by phosphorylation modifications. In plants, the CNGC consists of six transmembrane (TM) structural domains that form the channel pore region, a calmodulin-binding structural domain (CAMB) responsible for Ca^2+^/calmodulin-dependent regulation, and a cyclic nucleotide-binding structural domain (CNBD) that mediates cyclic nucleotide signaling, whereby the CNBD contains a critical phosphate-binding cassette (PBC) that is responsible for recognizing and binding cyclic nucleotide signaling molecules (e.g., cAMP and cGMP), whereas the hinge region adjacent to it is involved in the regulation of conformational changes. Calreticulin-binding cation transporter proteins were identified for the first time in 1998, when Schuurink et al. identified calreticulin-binding cation transporter proteins in barley (*Hordeum vulgare* L.) [8]. Subsequently, the CNGC family has been widely reported in many plant species, including *Arabidopsis thaliana* [9], rice [10], cotton [11], soybean [12], tomato [13], tobacco [14], oilseed rape [15], maize [16], wheat [17], upland cotton [18], and moso bamboo [19]. Based on the phylogenetic analyses of the above plants, it was shown that the plant CNGC gene family can be divided into four highly conserved evolutionary taxa (Classes I, II, III, and IV), of which Class IV can be further subdivided into two distinctly characterized subclasses (IV-a and IV-b).

The plant CNGC family is not only characterized by conserved structures but also extensively involved in a variety of key biological processes. Studies have shown that CNGCs play multiple regulatory roles in plant growth, development, and response to biotic and abiotic stresses. In terms of growth and development, Arabidopsis *AtCNGC1* is involved in a variety of physiological processes by mediating Ca^2+^ uptake, and several CNGC members in Arabidopsis synergistically regulate the morphogenesis of root hairs, with *AtCNGC5*, *AtCNGC6*, *AtCNGC9*, and *AtCNGC14* shown to play key roles in this process [20]. Further studies have shown that AtNGC14 has a unique regulatory function on the polar extension of root hairs by maintaining the cellular integrity of root hair apical growth. *AtCNGC14* loss-of-function mutants exhibit markedly abnormal root hair development. During plant sexual reproduction, CNGC is involved in pollen tube guidance by precisely regulating calcium ion signaling [21]. It has been shown that *AtCNGC16* and *AtCNGC18* play a role in pollen fertility and pollen tip growth under high temperature conditions, respectively, and mutations in genes such as *AtCNG16*/*AtCNGC18* lead to defective pollen tube development and reduced fertility [6,22]. The *OsCNGC13* gene enhances fertility in rice by promoting pollen tube growth in the pistil tissue [23].

In addition, the CNGC gene family plays important roles in response to different biotic and abiotic stresses. Arabidopsis *AtCNGC10* is involved in regulating salt tolerance in Arabidopsis. Mature plants of *AtCNGC10* antisense suppressor lines showed higher sensitivity to salt stress with a significant increase in aboveground Na^+^ accumulation compared to the wild type [24]. Knockdown of the cotton *GhCNGC32* and *GhCNGC35* genes decreased salt tolerance in cotton, which correlates with elevated expression of ABA-related genes under salt stress [11]. In rice, *OsCNGC9* regulates its channel activity through phosphorylation modification mediated by the SnRK2 family protein kinase *OsSAPK8*, which in turn promotes intracellular Ca^2+^ endocytosis during the seedling stage and ultimately enhances the cold tolerance of the plant [25]. In wheat, *TaCNGC14* and *TaCNGC16* negatively regulate plant disease resistance [17]. In tomato plants, *SlCNGC7* and *SlCNGC14* were shown to be involved as negative regulators in plant drought stress response. Compared with the wild type, *SlCNGC7* and *SlCNGC14* loss-of-function mutants exhibited enhanced drought tolerance, with a significant increase in leaf relative water content and a significant decrease in electrolyte leakage, suggesting that these two genes may negatively regulate drought tolerance in tomato through the regulation of ionic homeostasis [26].

Although the CNGC family in major cereals such as rice, maize, and wheat has been systematically identified, with some members confirmed to participate in processes like salt tolerance and drought resistance, understanding of the CNGC family in other cereals with unique stress resistance (such as sorghum) remains very limited. Therefore, we conducted a systematic identification, evolutionary analysis, and expression analysis of the CNGC family across the sorghum genome. This work aims to fill critical knowledge gaps in CNGC research between sorghum and other major cereals. Compared to rice, maize, and wheat, sorghum is renowned for its exceptional stress tolerance. Analyzing its SbCNGC family not only helps reveal the molecular basis of sorghum’s robust stress tolerance at the calcium signaling level but also enables the identification, through cross-species comparisons, of key CNGC members that are conserved in stress-tolerant species or unique to sorghum. These members hold promise as valuable genetic resources for future crop stress-tolerance genetic improvement.

## 2. Materials and Methods

### 2.1. Identification of Sorghum CNGC Gene Families

The reference genome and annotation files for the BTx623 sorghum variety were obtained from the NCBI database (https://www.ncbi.nlm.nih.gov/ (accessed on 10 June 2025)). Concurrently, the identified CNGC protein sequences in Arabidopsis were acquired from Tair (https://www.arabidopsis.org/ (accessed on 10 June 2025)) for comparative analysis. A Blastp comparison was conducted utilizing Tbtools (v2.322) software to identify possible CNGC homologous proteins within the sorghum protein library, employing Arabidopsis CNGC protein sequences as a reference and applying default filtering criteria. The initially acquired candidate sequences were subsequently filtered by the Pfam database (https://www.ebi.ac.uk/interpro/entry/pfam (accessed on 10 June 2025)) to keep those having CNGC structural domains (PF00520 and PF00027). HMMs were developed with the HMMER (v3.3.2) tool to enhance accuracy and conduct Hidden Markov Model (HMM) searches on sorghum protein sequences to identify probable CNGC family members. Results from Blastp (2.13.0) and HMMER analyses were cross-referenced, and redundant sequences were removed using the CD-HIT (v4.8.1) tool to ensure the identification of unique, high-quality candidate protein sequences. Based on their top-down chromosomal arrangement, the genes were sequentially named SbCNGC1 through SbCNGC23.

### 2.2. Analysis of SbCNGC Chromosome Localization and Physicochemical Properties

Extract the SbCNGC chromosome location information from the sorghum reference genome annotation file and visualize it using the Tbtools software. Protein physicochemical properties were determined using the ExPasy website (https://web.expasy.org/protparam/, accessed on 15 June 2025). Subcellular localization prediction was performed using the Cell-PLoc 2.0 tool (http://www.csbio.sjtu.edu.cn/bioinf/plant-multi/, accessed on 15 June 2025).

### 2.3. Phylogenetic Analysis of CNGC in Sorghum

Download CNGC protein sequences from *A. thaliana*, *Oryza sativa*, *Zea mays*, *H. vulgare*, and *Saccharum spontaneum* using the Ensembl Plant database (https://plants.ensembl.org/index.html (accessed on 15 June 2025)), NCBI database (https://www.ncbi.nlm.nih.gov/ (accessed on 15 June 2025)), and Tair (https://www.arabidopsis.org/ (accessed on 15 June 2025)). Phylogenetic studies of the CNGC protein amino acid sequences for these species were conducted utilizing the default parameters of the Neighbour-Joining (NJ) algorithm in the MEGA 11 software, with Bootstrap values established at 1000 replicates. Phylogenetic trees were developed to compare sorghum with Arabidopsis, rice, maize, barley, and wild sugarcane. The phylogenetic trees were visualized using the online platform ITOL (https://itol.embl.de/login.cgi (accessed on 17 June 2025)), and the graphics were further enhanced and refined with Adobe Illustrator 2022 (v26.4.1).

### 2.4. SbCNGC Gene Characteristics

We visualized the gene structures of SbCNGC family members using the “Gene Structure Visualization” feature in TBtools (v2.322). To predict conserved motifs in SbCNGC proteins, we locally ran MEME Suite (v2.322) within TBtools and analyzed all SbCNGC protein sequences. Additionally, conserved domains in the proteins were predicted using the NCBI-CDD tool (https://www.ncbi.nlm.nih.gov/, accessed on 17 June 2025). To further analyze the secondary structure of SbCNGC proteins, we employed the NPSA platform (https://npsa.lyon.inserm.fr/cgi-bin/npsa_automat.pl?page=/NPSA/npsa_sopma.html, accessed on 17 June 2025). Finally, a three-dimensional structural model of the SbCNGC protein was constructed using the SWISS-MODEL website (https://swissmodel.expasy.org/, accessed on 20 June 2025).

### 2.5. Predictive Analysis of Cis-Elements of the SbCNGC Promoter

Using the TBtools tool, we extracted the promoter sequences spanning 2000 bp upstream of the start codon for the SbCNGC gene. We uploaded these sequences to the PlantCARE online database (http://bioinformatics.psb.ugent.be/webtools/plantcare/html/, accessed on 20 June 2025) for screening cis-regulatory elements [27,28]. After excluding core promoter elements and components with unidentified functions, we performed quantitative analysis on the identified regulatory elements. Subsequently, visualization was conducted via the TVBOT website (https://www.chiplot.online/tvbot.html, accessed on 23 June 2025).

### 2.6. Duplication Analyses of SbCNGCs

Gene duplication events within the sorghum SbCNGC family were analyzed and visualized using TBtools, which includes its Circos module. The selective pressure on these events was subsequently assessed by calculating the Ka/Ks ratio for each duplicated gene pair. To investigate evolutionary conservation, genomic and annotation data for seven plant species—*A. thaliana*, *O. sativa*, *Z. mays*, *H. vulgare*, *Glycine max*, *Setaria italica*, and *Arachis hypogaea*—were retrieved from NCBI. MCScanX was then employed to identify orthologous SbCNGC gene pairs between sorghum and these species, thereby facilitating interspecies comparative analysis.

### 2.7. Prediction of miRNAs Targeting SbCNGC

To predict miRNAs that may regulate members of the sorghum SbCNGC gene family. The CDS of all SbCNGC genes were first extracted using Tbtools. Subsequently, the sequences were submitted to the online tool psRNATarget (https://www.zhaolab.org/psRNATarget/ (accessed on 25 June 2025)) and analyzed using default parameters to identify potential miRNA-target gene interactions. Finally, the Origin 2025 software visualized the miRNA-target relationship with SbCNGC to investigate the potential miRNA regulatory roles further.

### 2.8. GO and KEGG Annotation Analysis

The SbCNGC family members were annotated with GO function and KEGG pathway using the eggNOG-mapper (http://eggnog5.embl.de/#/app/home (accessed on 28 June 2025)) online web tool to obtain the annotation result files in TSV format—the latest go-basic. For subsequent analysis, an OBO file was also downloaded from the Gene Ontology (https://geneontology.org/ (accessed on 28 June 2025)) database. Using TBtools software, 23 SbCNGC genes were enriched and analyzed using the software’s built-in GO enrichment analysis tool and KEGG pathway enrichment analysis tool. The GO and KEGG annotation analysis revealed the functional features of the SbCNGC gene family and the biological processes they may be involved in, which provided important clues for an in-depth understanding of the function of this gene family.

### 2.9. Analysis of SbCNGC Gene Expression Pattern in Different Tissues and Different Abiotic Stresses

RNA-seq data were obtained from public repositories to analyze SbCNGC expression across developmental tissues and abiotic stress conditions. Data under project PRJEB22168, sourced from the European Nucleotide Archive (https://www.ebi.ac.uk/ena/browser/home (accessed on 3 July 2025), were used to profile expression across seven distinct tissue types: root, stem, seedling, endosperm, inflorescence, pollen, and pericarp. For stress-responsive expression analysis, data under project PRJNA1159475 were retrieved from the NCBI SRA database (https://www.ncbi.nlm.nih.gov/ (accessed on 3 July 2025). This dataset encompasses transcriptomes from sorghum seedlings subjected to heat stress (42 °C) and cold stress (4 °C) over a time-course experiment. Gene expression levels were quantified using FPKM values and standardized via log2(FPKM + 1) transformation to approximate a normal distribution. The standardized expression matrix was visualized as a heatmap using TBtools software.

### 2.10. Plant Material and Abiotic Stress Treatments

This study employed the sorghum cultivar Ji 2055B. Prior to sowing, seeds were disinfected with 75% ethanol and rinsed with sterile water. Seedlings were cultivated in a growth chamber (GZLG-P280C, Hefei Daskate Biotechnology Co., Ltd., Hefei, China) using a substrate mixture of peat moss, perlite, and vermiculite (3:1:1) under controlled conditions: 25 °C, 12 h light/12 h dark, 5000 lux, and 60–70% relative humidity. After reaching the three-leaf stage, plants were subjected to either low-temperature (4 °C) or high-temperature (42 °C) stress treatments. Leaf samples were collected at 0, 6, 12, and 24 h post-treatment initiation, immediately flash-frozen in liquid nitrogen, and stored at −80 °C. Three biological replicates were established for each treatment group and time point.

### 2.11. Real-Time Quantitative PCR (qPCR) Analysis of SbCNGC Gene Expression Under Cold and Heat Stresses

Total RNA was extracted from sorghum using the SteadyPure Plant RNA Quick Extraction Kit (Accurate Biotechnology, AG21040, Changsha, China) according to the manufacturer’s instructions. Subsequently, cDNA was synthesized by reverse transcription using the Evo M-MLV RT Mix Kit with gDNA Clean for qPCR Ver. 2 (Accurate Biotechnology, AG11728, Changsha, China) and stored at −20 °C until use. Gene-specific primers were designed and synthesized by Sangyo Bioengineering (Shanghai, China) Co., Ltd. (see Supplementary Material Table S1). Quantitative real-time PCR (qPCR) was performed using the SYBR Green Premix Pro Taq HS qPCR Kit (Accurate Biotechnology, AG11718, Changsha, China) on a cDNA template. The 20 μL reaction mixture consisted of 10 μL of 2× SYBR Green Pro Taq HS Premix, 2 μL of diluted cDNA, 0.4 μL of each forward and reverse primer, and nuclease-free water. The thermal cycling conditions were as follows: initial denaturation at 95 °C for 30 s, followed by 40 cycles of denaturation at 95 °C for 5 s and annealing/extension at 60 °C for 30 s. The EIF4α gene was used as an internal reference for normalization. All experiments included three biological replicates with three technical replicates each. Relative gene expression levels were calculated using the 2^−ΔΔCT^ method.

## 3. Results

### 3.1. Genome-Wide Identification of the SbCNGC Gene in Sorghum

**Figure 1:**
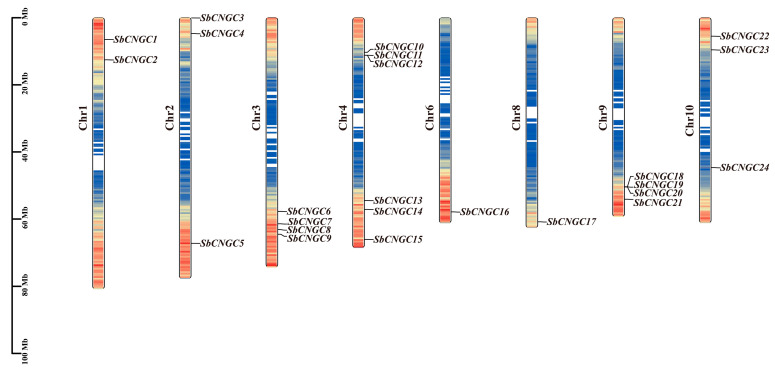
Chromosomal distribution of the sorghum SbCNGC gene family. Chromosome numbers are labeled in black. The names and specific locations of SbCNGC genes are indicated in blue. The physical lengths of the chromosomes are shown by the scale on the left. The color gradient inside the chromosomes represents gene density, ranging from red (high density) to blue (low density).

This study used a combination of protein sequence-based BLASTp comparison and HMMER analysis. Twenty-three *SbCNGC* genes were successfully identified from the sorghum genome and named *SbCNGC1* to *SbCNGC23* based on their physical locations on the chromosomes (see Table 1). The distribution of *SbCNGC* genes on the chromosomes was heterogeneous, with the genes being distributed on chromosomes 1, 2, 3, 4, 6, 8, 9, and 10 (Figure 1), with no *SbCNGC* genes detected on chromosomes 5 and 7 (Figure 1). No *SbCNGC* gene family members were detected on chromosomes 5 and 7. Chromosome 4 had the highest number of *SbCNGC* genes, containing six, followed by chromosomes 3 and 9, each containing four *SbCNGC* genes. In contrast, chromosomes 6 and 8 contained only one gene each. Regarding protein physicochemical properties, the 23 SbCNGC proteins varied greatly in length. Among them, *SbCNGC3* encoded the bulkiest protein, containing 919 amino acid residues, while *SbCNGC7* was the shortest, containing only 530 amino acids. Molecular mass analysis showed that the relative molecular mass of *SbCNGC7* was approximately 61 kDa, while that of *SbCNGC3* was approximately 101 kDa. Additionally, isoelectric point (pI) analysis revealed that all SbCNGC proteins exhibited pI values ranging from 5.96 (*SbCNGC22*) to 9.74 (*SbCNGC20*), with an average of 8.34, indicating that most SbCNGC proteins are more stable under weakly alkaline conditions. Further hydrophilicity/hydrophobicity analysis revealed that nearly all SbCNGC proteins are hydrophilic, with *SbCNGC1*, *SbCNGC6*, *SbCNGC10*, and *SbCNGC12* exhibiting slight hydrophobicity. This property may correlate with their involvement in cell membrane-associated signaling and ion transport processes. Subcellular localization predictions indicate that most SbCNGC proteins (17) are primarily localized to the cell membrane, consistent with their function as ion channel proteins. However, six protein-coding genes (including *SbCNGC3*, *SbCNGC5*, *SbCNGC17*, *SbCNGC18*, *SbCNGC19*, and *SbCNGC22*) are predicted to be localized to the cell nucleus.

### 3.2. Phylogenetic Analysis of the SbCNGC Gene Family

**Figure 2:**
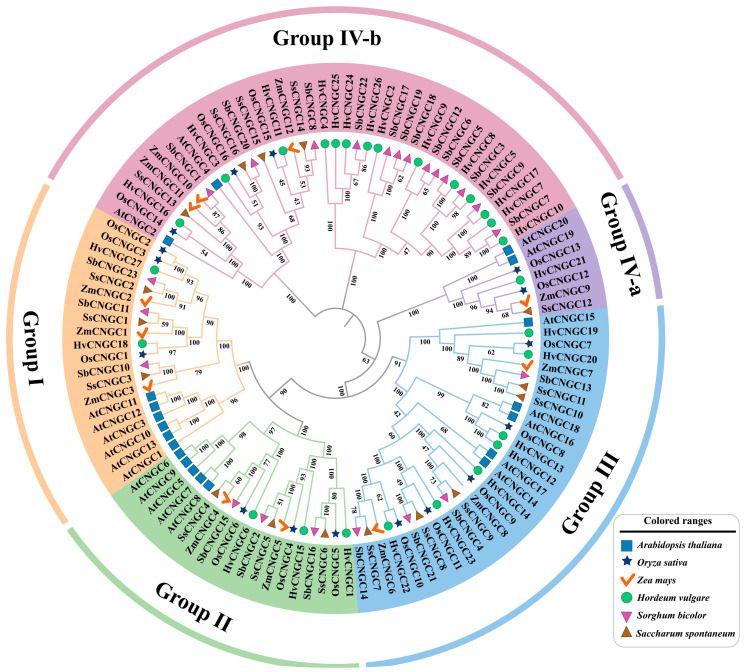
Phylogenetic analysis of CNGC family members (*A. thaliana*, *O. sativa*, *Z. mays*, *H. vulgare*, *S. bicolor*, and *S. spontaneum*). Different colored shapes represent different species.

In order to profoundly investigate the evolutionary relationship and phylogenetic features of the SbCNGC gene of Sorghum with CNGC genes in other plants, the sequences of CNGC proteins in *Sorghum bicolor*, *A. thaliana*, *O. sativa*, *Z. mays*, *H. vulgare*, and *S. spontaneum*, totalling 114 members of CNGC protein sequences, were subjected to multiple sequence comparisons using the MEGA software, and a phylogenetic tree was constructed by the Neighbour-Joining (NJ) method (Figure 2). The phylogenetic tree was constructed following the standard classification of *A. thaliana* as a reference, and all CNGC genes were classified into five major evolutionary branches: Group I, Group II, Group III, Group IV-a, and Group IV-b. The Group I class contains 20 CNGC members (6 AtCNGCs, 3 OsCNGCs, 3 ZmCNGCs, 2 HvCNGCs, 3 SsCNGCs, and 3 SbCNGCs); the Group II class contains 19 CNGC members (5 AtCNGCs, 3 OsCNGCs, 2 ZmCNGCs, 3 HvCNGCs, 3 SsCNGCs, and 3 SbCNGCs). Group III class contains 29 CNGC members (5 AtCNGCs, 5 OsCNGCs, 3 ZmCNGCs, 7 HvCNGCs, 5 SsCNGCs, and 4 SbCNGCs). Class Group IV-a contains 7 CNGC members (2 AtCNGCs, 2 OsCNGCs, 1 ZmCNGC, 1 HvCNGC, and 1 SsCNGC). Notably, the SbCNGC members of Sorghum were not detected in this taxon, suggesting that this class of CNGCs may have undergone deletions or functional substitutions in Sorghum. Group IV-b contains 39 CNGC members (2 AtCNGC, 3 OsCNGC, 3 ZmCNGC, 14 HvCNGC, 4 SsCNGC, and 13 SbCNGC). From the distribution results of the phylogenetic tree, the sorghum SbCNGC gene family members were more widely distributed in various evolutionary branches. They showed significant enrichment in Group IV-b, suggesting that this class of genes may have unique physiological functions and adaptive evolutionary features in Sorghum.

### 3.3. Gene Structure and Conserved Motifs of SbCNGC Genes

**Figure 3:**
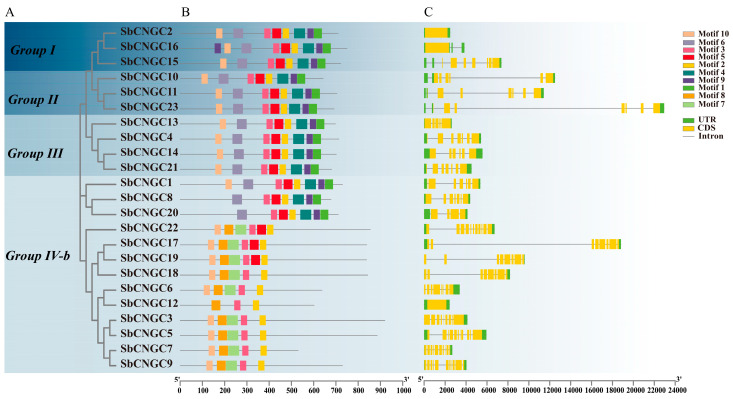
Structural analysis of the SbCNGC gene. Phylogenetic tree and grouping (**A**); motif sequence (**B**); (**C**) gene exon/intron structure.

**Figure 4:**
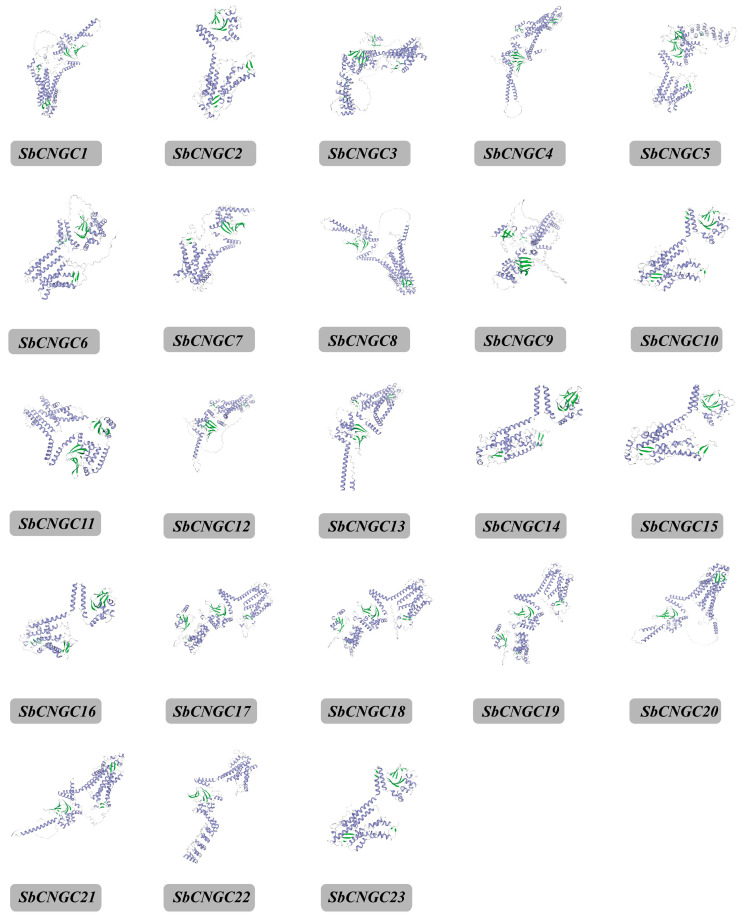
SbCNGC protein tertiary structure (alpha helix (purple), Beta turn (green) and Random coil (grayish white)).

The structural features of the *SbCNGC* gene family of sorghum and its functional conservation were resolved by performing motif prediction analysis of the *SbCNGC* gene family using the MEME online website (Figure 3). The results showed that the SbCNGC family encodes proteins with 10 conserved motifs (3B). Members of the same subfamily exhibit similar distribution patterns of these conserved motifs. Among all SbCNGC proteins, Motif2 and Motif3 are the most conserved motifs, present in all members, suggesting that they may play a key role in maintaining the core function of CNGC proteins. In addition, Motif10 was absent in *SbCNGC8* and *SbCNGC20*, suggesting that these members may have undergone structural or functional specialization or simplification. To assess the structural diversity among SbCNGC genes, we examined their exon-intron organization by comparing their coding sequences (CDS) with the corresponding genomic sequences. The analysis revealed that most SbCNGC family members contain introns, with considerable variation in intron numbers across different phylogenetic groups. Specifically, Group I members (3 genes) harbored 0 to 8 introns, including the intronless *SbCNGC2*. Groups II and III exhibited 6–7 and 4–6 introns in their three and four members, respectively. The most pronounced diversity was observed in Group IV-b, where its 13 members contained between 0 and 10 introns. Although there was some variation in the number of introns, the exon-intron arrangement of members within the same subgroup was generally relatively consistent. It showed high structural conservation, reflecting their evolutionary homology and functional convergence. Additionally, analysis of the protein’s secondary structure (see Supplementary Material Table S2) and tertiary structure indicates that SbCNGC is primarily composed of α-helices and irregularly coiled chains (Figure 4).

### 3.4. Comparative Analysis of SbCNGC Gene Duplication Events with Other Species

**Figure 5:**
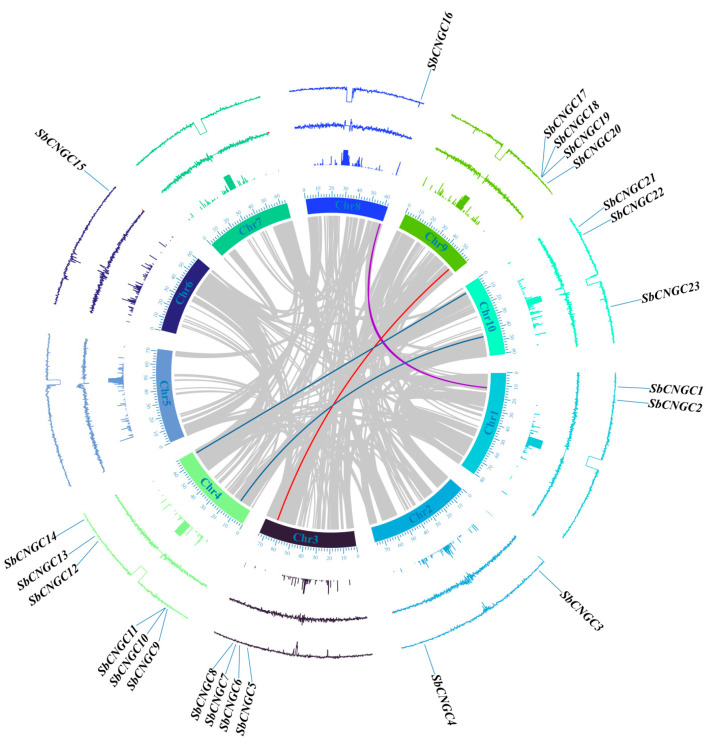
Results of intraspecific covariance analysis of the sorghum *SbCNGC* gene family. In order from the inside out: chromosome number, unknown base distribution, GC content deviation, leaf transcriptome read segments, and GC content. Red, blue, and purple curves connecting *SbCNGC* genes indicate duplicate gene pairs in the sorghum *SbCNGC* gene family.

**Figure 6:**
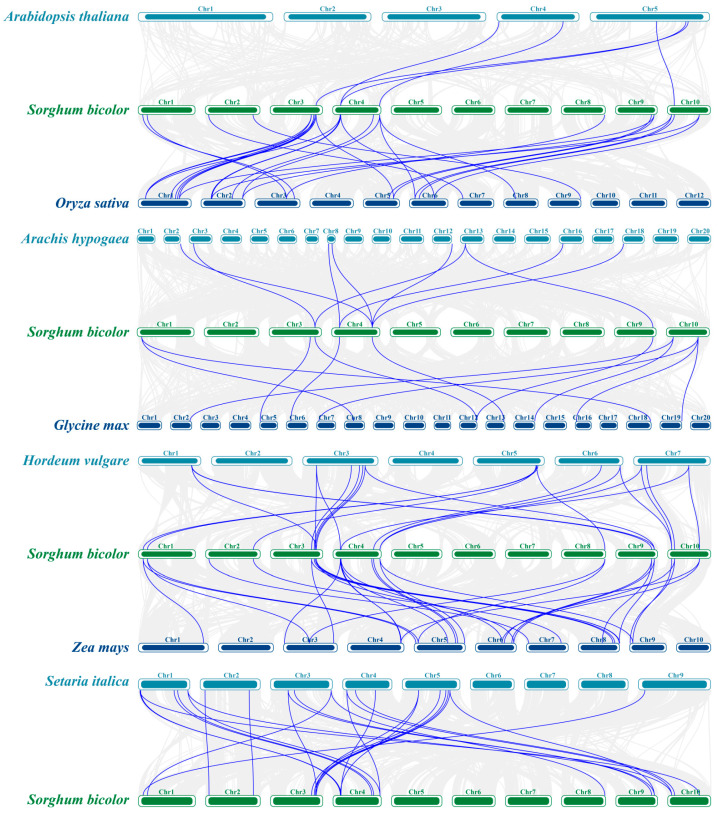
Co-linearity analysis of sorghum with *SbCNGC* genes from seven different plants. Gray lines in the background indicate blocks of sorghum repeating events with *A. thaliana*, *O. sativa*, *A. hypogaea*, *G. max*, *H. vulgare*, *Z. mays*, and *S. italica*, and the blue line highlights covariates as gene pairs homologous to SbCNGC.

To investigate the origin of the *SbCNGC* gene family and its evolutionary dynamics, we analyzed gene duplication events in SbCNGC. Four tandem or segmentally duplicated gene pairs were identified among the 23 SbCNGC family members (Figure 5), suggesting that this gene family may have undergone a localized gene duplication expansion in sorghum as one of the important drivers of its diversity formation. To better understand the homology and evolutionary relationships between *SbCNGC* genes and other different species, we selected common monocotyledonous plants (Gramineae-dominated), *O. sativa*, *Z. mays*, *S. italica*, and *H. vulgare*, and dicotyledonous plants (Leguminosae and Cruciferae), *A. thaliana*, *G. max*, and *A. hypogaea.* We analyzed them by covariance analysis to compare their CNGC homology (Figure 6). The results of the repeating event comparison showed that sorghum *SbCNGC* genes showed high homology in comparison with monocotyledonous plants. Twenty-six, 32, 25 and 21 pairs of homologous genes were found in comparisons between sorghum and rice, maize, cereal and barley CNGCs, respectively, in monocotyledonous plants; in contrast, the covariance between sorghum and dicotyledonous plants was significantly weaker. Comparisons between sorghum and *A. thaliana*, soybean and peanut CNGCs found 5, 12 and 9 pairs of homologous genes, respectively, among dicotyledonous plants. This result indicates that *CNGC* genes are highly conserved and homologous between sorghum and other gramineous monocotyledons. In particular, the covariance was closer in crops with closer relatives, such as maize and rice, reflecting their high phylogenetic relevance. On the contrary, the number of co-lined genes with dicotyledons was significantly reduced, suggesting that the CNGC family may have undergone different evolutionary pathways and functional differentiation processes in both monocotyledonous and dicotyledonous lineages.

**Figure 7:**
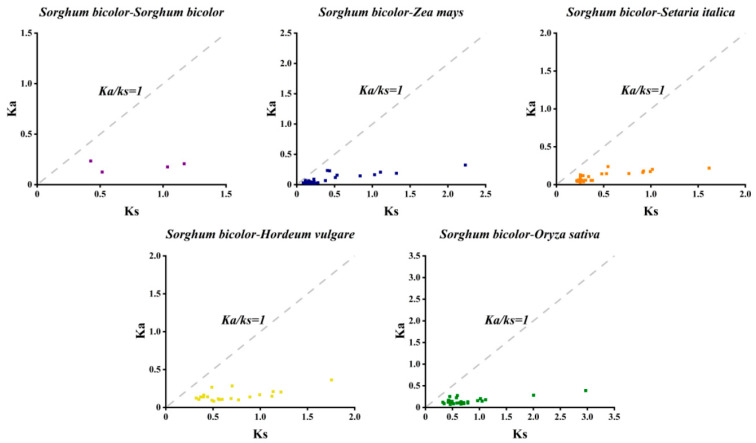
Scatter plot of Ka and Ks for CNGC homologous gene pairs in sorghum and monocotyledonous (grass-dominated) plants, with the *x*-axis denoting Ks and the *y*-axis denoting Ka.

To assess the selection pressure on the sorghum *SbCNGC* gene family during evolution, homologous gene pairs duplicated within sorghum itself and between sorghum and other grasses (including *O. sativa*, *Z. mays*, *H. vulgare* and *S. italica*), between CNGC homologous gene pairs and their Ka/Ks ratios were calculated (Figure 7). The analysis showed that the Ka/Ks ratio was less than 1 in all compared homologous *CNGC* gene pairs without exception. This suggests that, due to *CNGC* gene duplication events within sorghum and homologous gene relationships with other monocotyledons, the sorghum CNGC family has been subjected to significant purifying selective pressures during evolution.

### 3.5. Analysis of Cis-Acting Elements in the Promoter Regions of SbCNGC Genes

**Figure 8:**
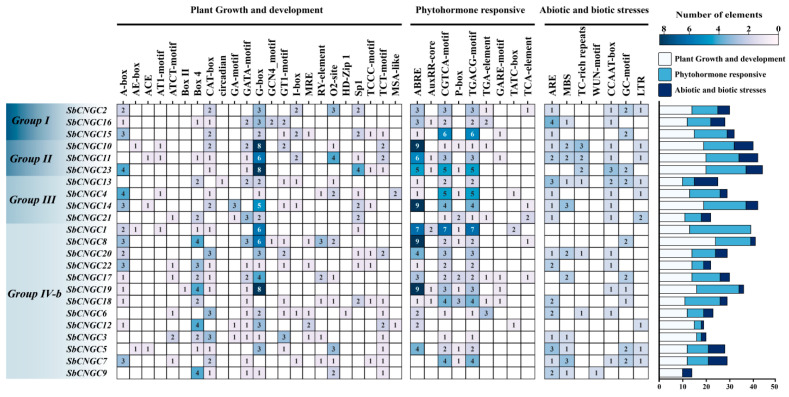
Analysis of cis-acting elements in the SbCNGC promoter.

To predict the transcriptional regulatory mechanism of the SbCNGC gene in sorghum, twenty-three promoter sequences of the 2000 bp region upstream of the SbCNGC gene were extracted using TBtools, and the Plants CARE database was analyzed for their cis-acting elements. A total of 39 different types of cis-acting elements were detected in all promoter sequences (Figure 8), which could be broadly categorized into three groups according to their functions: plant growth and development-related elements (23 types), hormone response-related elements (9 types), and abiotic and biotic stress response-related elements (7 types).

Among the plant growth and development-related cis-acting elements, light-responsive elements (G-Box and Box 4) were the most abundant, with 78 and 33 occurrences, respectively, suggesting that light conditions may regulate the expression of SbCNGC genes. Among the hormone response-related cis-acting elements, a variety of phytohormone response elements were widely detected in the promoter region, including abscisic acid, jasmonic acid, gibberellins, salicylic acid, and growth factors. Among them, abscisic acid and jasmonic acid (CGTCA and TGACG elements) response elements were more significant, appearing 80 times (ABRE), 63 times (CGTCA), and 63 times (TGACG element), respectively, suggesting that the SbCNGC gene may be susceptible to the abscisic acid and jasmonic acid hormone signaling pathway. Presumably, it may play an important role in plant adversity stress signaling. In addition, the SbCNGC promoter was enriched with a variety of stress-associated elements, such as the anaerobic-inducible elements ARE (29 times) and GC (18 times), the low-temperature-responsive element LTR (10 times), the drought-inducible element MBS (19 times), the defense- and stress-associated elements TC (10 times) and CCAAT-box (17 times), and the wound-responsive element WUN-motif (1 time). These cis-acting elements indicate that the *SbCNGC* gene family may play an important role in sorghum’s response to various abiotic and biotic stresses.

### 3.6. miRNA-Mediated Post-Transcriptional Regulation of SbCNGC Genes

**Figure 9:**
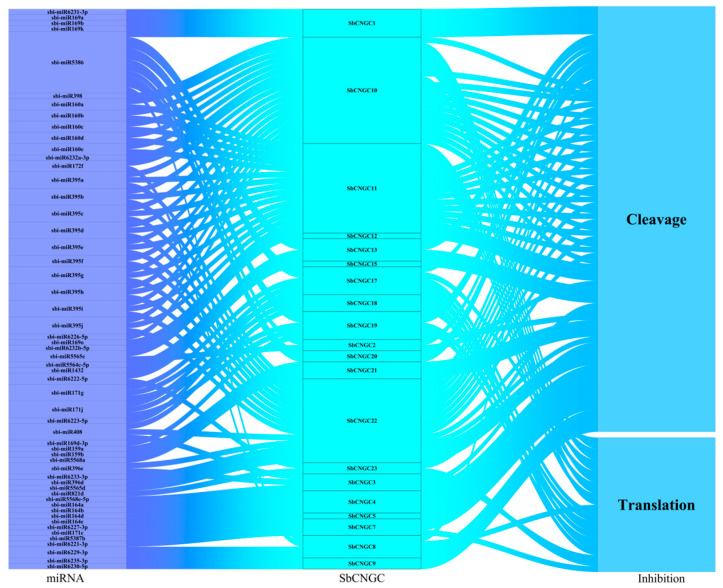
Sankey plot of miRNA targeting in relation to SbCNGC transcripts. The three columns represent miRNA, mRNA, and repression. Each rectangle represents a gene, and the connectivity of each relationship is visualized based on the size of the corresponding rectangle.

As shown in Figure 9, the results indicate that multiple miRNA families may be involved in regulating SbCNGC gene expression. Among them, the miR395 and miR160 families were particularly prominent: all 10 members of miR395 targeted *SbCNGC10*, *SbCNGC11* and *SbCNGC22*, inhibiting target gene expression through the cleavage mechanism, whereas the five members of miR160 regulated the same target genes through translational repression, suggesting that these two miRNA families may play a central regulatory role in the calcium signaling pathway. In addition, miR5386 showed extensive multi-targeting, targeting six different SbCNGC genes simultaneously with cleavage and translational repression mechanisms, suggesting that it may act as a key node to integrate multiple signaling pathways. Notably, some miRNA binding sites were highly conserved in target genes; for example, the miR160 family recognizes identical sequences in the CDS regions of *SbCNGC10* and *SbCNGC11*, which may reflect functional evolutionary conservation. These findings provide new clues for understanding the miRNA-mediated CNGC gene regulatory network in plants and, in particular, lay the foundation for investigating the mechanism of calcium signaling-related stress response.

### 3.7. GO Enrichment and KEGG Pathway Analysis of SbCNGC Gene Family Members

**Figure 10:**
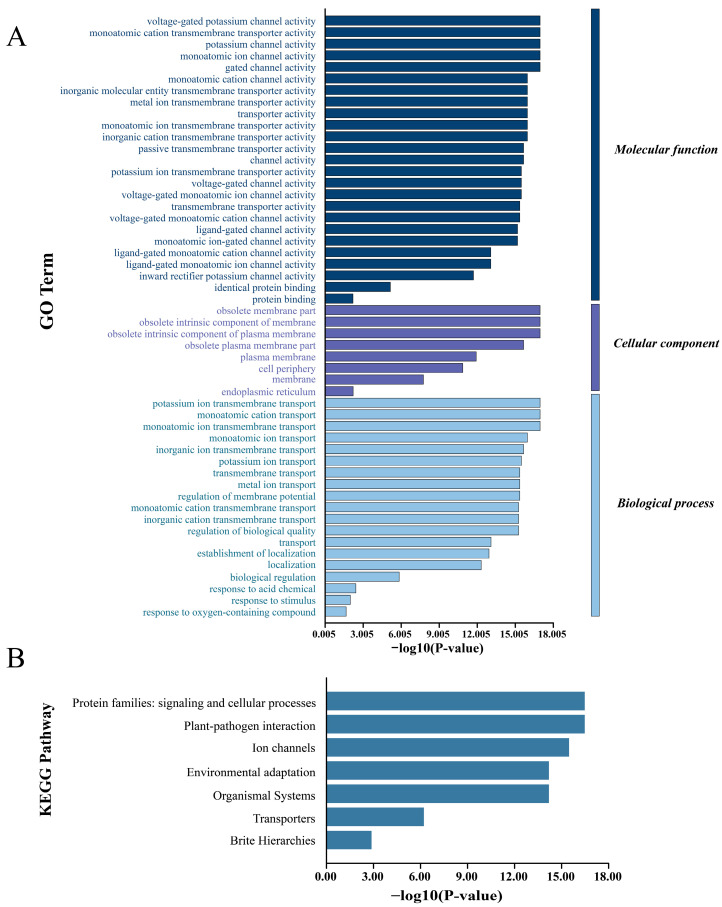
GO enrichment (**A**) and KEGG (**B**) analysis of *SbCNGC* gene family members.

To further elucidate the biological roles of the sorghum SbCNGC gene family at the functional and signaling pathway levels, this study conducted Gene Ontology (GO) functional enrichment analysis and KEGG pathway annotation analysis. Based on the GO enrichment results (Figure 10A), SbCNGC family members exhibit significant functional and biological associations with potassium channel function and ion transport processes. At the molecular function level, voltage-dependent ion channel activity and potassium ion transmembrane transport activity exhibited the highest enrichment scores, indicating that this gene family plays a crucial role in regulating voltage-dependent ion channel activity and selective potassium ion transport. At the cellular component level, SbCNGC family members were primarily enriched in the plasma membrane, which was consistent with their transmembrane ion transport function. Regarding biological processes, potassium ion transmembrane transport and membrane potential regulation were significantly enriched, further confirming the important role of SbCNGC genes in electrophysiological regulation. As shown in Figure 10B, SbCNGC was significantly enriched in several key signaling pathways and biological processes. Among them, the ion channel pathway showed the strongest enrichment significance, highly consistent with the voltage-gated potassium channel activity found in GO analysis, further confirming the central role of SbCNGC family members in electrical signaling and ion transmembrane transport. In addition, the transporter protein pathway likewise showed significant enrichment, suggesting that these genes may be involved in a broader range of substance transport processes.

### 3.8. Expression Profiles of SbCNGC Genes in Different Tissues

**Figure 11:**
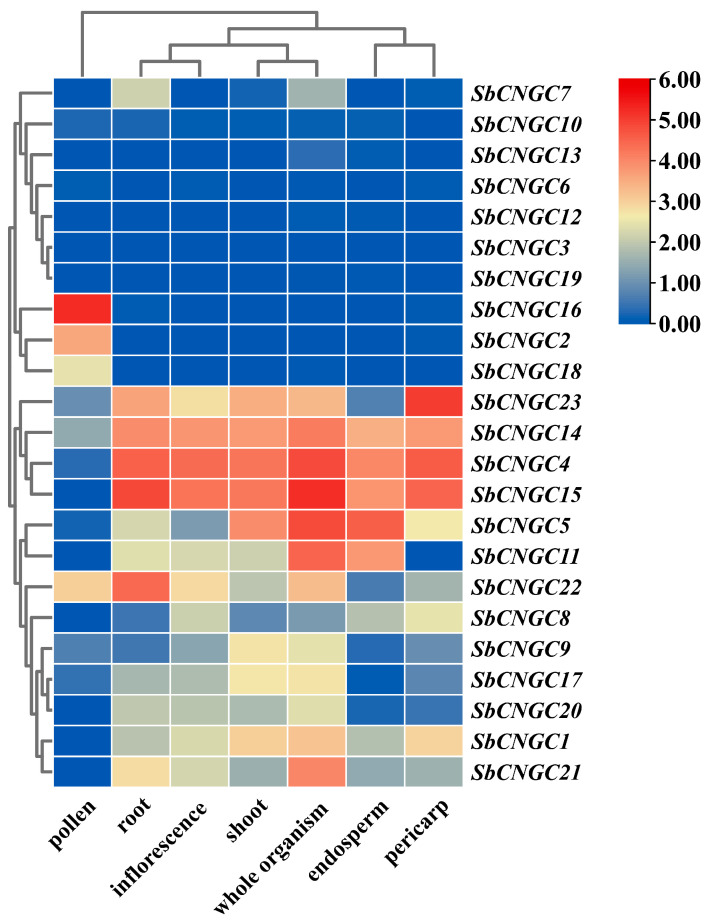
Tissue-specific expression profiles of sorghum *CNGC* gene family members.

In order to gain insights into the potential biological functions of Sorghum *CNGC* gene family members, a systematic analysis of the expression patterns of *SbCNGC* genes in different tissues was conducted by using transcriptomic data to reveal the possible functional differentiation of the *SbCNGC* genes in different tissues, such as roots, shoots, endosperm, seedlings, pollen, inflorescences and pericarp (Figure 11). The results showed that several tissues expressed most SbCNGC genes to different degrees. However, there were significant differences in their tissue-specific expression patterns, reflecting their possible functional differentiation in different physiological processes. Specifically, *SbCNGC4*, *SbCNGC14*, and *SbCNGC15* showed high expression in several tissues, including root, shoot, endosperm, seedling, pollen, and inflorescence. This suggests that *SbCNGC* genes play important roles in plant growth and development, primarily regulating key physiological processes such as root development, stem and leaf extension, and inflorescence formation. In contrast, the expression of *SbCNGC16* was low in roots, shoots, endosperm, seedlings, pollen, and inflorescence but significantly increased in pollen. In addition, the genes *SbCNGC2*, *SbCNGC3*, *SbCNGC6*, *SbCNGC10*, *SbCNGC13*, *SbCNGC12*, and *SbCNGC19* were expressed at lower levels in several tissues. This suggests that these genes may be conditionally expressed, depending on developmental stage or environmental stimuli.

### 3.9. Expression Analysis of SbCNGC Genes Under Abiotic Stresses

**Figure 12:**
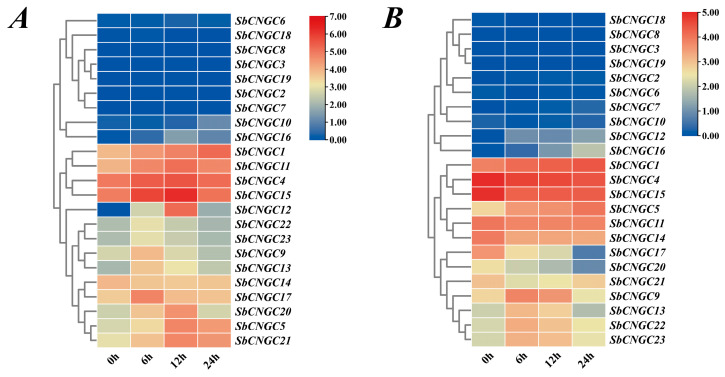
Expression profiles of sorghum *CNGC* gene family members under abiotic stress. Low temperature (**A**); high temperature (**B**).

To investigate the response mechanisms of the SbCNGC gene family members under various abiotic stresses, this study analyzed their expression patterns across different abiotic stress conditions, including low temperature, high temperature, drought, and salt stress, using transcriptome data from public databases (NCBI and ENA). Using the 0 h time point as the control, expression trends at different time points were observed through time point analysis (Figure 12). Under cold stress, most genes (e.g., *SbCNGC6*, *SbCNGC18*, *SbCNGC8*, *SbCNGC3*, *SbCNGC19*, etc.) exhibited sustained low expression between 0 and 24 h, while a few genes (e.g., *SbCNGC11*, *SbCNGC4*, *SbCNGC15*, *SbCNGC12*) showed significant upregulation after 6–12 h, suggesting these genes may participate in cold signal response processes. Notably, the expression level of the *SbCNGC1* gene gradually increased over time, suggesting it may serve as one of the core Ca^2+^ channels for cold signal perception in plants. Under high-temperature stress, *SbCNGC1* expression progressively increased with treatment duration, exhibiting typical heat response characteristics; conversely, *SbCNGC4* and *SbCNGC15* expression levels continuously declined, suggesting that they may participate in maintaining plant cellular homeostasis through negative regulatory pathways. The above data indicate that SbCNGC genes exhibit distinct temporal specificity in their expression patterns under various stress conditions, and these genes perform diverse biological functions in response to environmental stresses, including low temperature, high temperature, drought, and salt stress.

### 3.10. Time-Specific Analysis of SbCNGC Gene Expression Under Cold and Heat Stress Conditions

**Figure 13:**
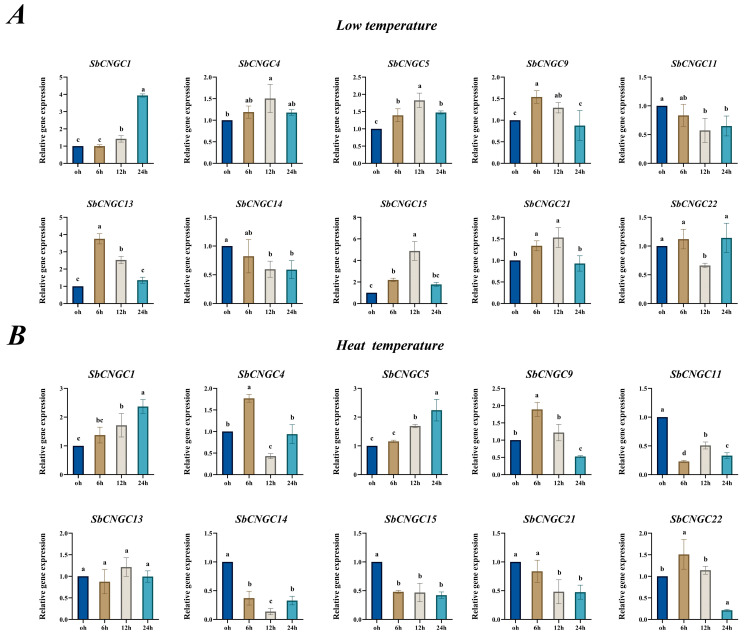
Relative expression profiles of SbCNGC genes under different stress tolerances. Low temperature (**A**); high temperature (**B**). Each bar represents the subject mean of 3 replicates, and error bars represent standard deviation (SD). Lowercase letters indicate differences significant at the 0.05 level.

To verify the accuracy and reliability of the transcriptome data, 10 *SbCNGC* gene members (including *SbCNGC1*, *SbCNGC4*, *SbCNGC5*, *SbCNGC9*, *SbCNGC11*, *SbCNGC13*, *SbCNGC14*, *SbCNGC15*, *SbCNGC21* and *SbCNGC22*) and their expression were detected by qRT-PCR at different treatment time points (0 h, 6 h, 12 h and 24 h) under cold stress and heat stress treatments (Figure 13). Results indicate that the expression trends of most genes at different time points align with transcriptomic data, further validating the reliability of transcriptomic analysis. Under low-temperature stress treatment, *SbCNGC5* and *SbCNGC15* were significantly upregulated at 6 h, demonstrating their capacity for rapid response to cold stress—a trend consistent with transcriptomic data. Concurrently, *SbCNGC1* expression levels gradually increased with treatment duration, suggesting its potential involvement in late-stage cold stress signaling or adaptive regulation. Under heat stress, *SbCNGC4*, *SbCNGC9*, and *SbCNGC22* rapidly peaked at 6 h before gradually declining, indicating their likely role in early heat stress responses followed by regulatory suppression to prevent plant overreaction. In contrast, *SbCNGC1* exhibited sustained upregulation under heat stress, demonstrating a stable thermal response pattern that further confirms its pivotal regulatory role in heat stress. Overall, *SbCNGC1* exhibits a sustained upward trend in both cold and heat stress treatments, demonstrating stable and persistent expression characteristics. This suggests that this gene plays critical regulatory functions in both cold and heat stress responses and may serve as an important regulator within the plant’s multistress signaling network.

## 4. Discussion

Cyclic nucleotide-gated channel (CSNGC) proteins, as an important component of the calcium signaling pathway, are widely found in plants and animals, especially in plants, where they are involved in several key physiological processes such as ion transport, signal sensing, stress response and growth regulation [29]. However, there are fewer studies on the *CNGC* gene family in sorghum, and its mechanism of action in plant development and abiotic stress response is unclear. In this study, we systematically identified and analyzed the structural features, evolutionary relationships, expression patterns and their responses to abiotic stresses of the *CNGC* gene family in sorghum by genomic and transcriptomic approaches, aiming to provide a theoretical basis for subsequent functional studies and breeding for stress tolerance.

First, a total of 23 *SbCNGC* genes were identified using sorghum genomic data, which were quantitatively intermediate between other plants such as *A. thaliana* 20 [24], rice 16 [30], maize 12 [16], and barley 27 [31], showing an inevitable expansion or contraction of the CNGC gene family in different plants. Such quantitative differences may be closely related to species-specific gene duplication, loss and functional differentiation. Chromosomal localization analysis revealed that *SbCNGC* genes were significantly clustered on chromosomes 4 and 9, suggesting that the family may have undergone gene expansion or local duplication events. Gene structure and conserved motif analysis showed that most *SbCNGC* genes had highly consistent exon-intron structures and conserved motif composition, and all contained typical CNGC structural domains. Phylogenetic tree analysis classified *SbCNGC* genes into five major taxa (I–IV-b), which was consistent with the classification in model plants such as *A. thaliana* and rice; however, no SbCNGC members were detected in Group IV-a, which may be a good example of how SbCNGC genes are categorized. SbCNGC members, possibly suggesting that sorghum has lost events or evolutionary pathway differences in this class of genes. Subcellular localization predictions indicated that most SbCNGCs were localized to the cell membrane and a few to the nucleus. This is similar to the previous study in maize *CNGC* gene family members [16]. The analysis of covariance showed that *SbCNGC* genes were strongly conserved between sorghum and monocotyledonous plants such as rice, maize, and barley.

In contrast, covariance with dicotyledonous plants (e.g., *A. thaliana*, soybean, and peanut) was weaker, indicating that the gene family underwent their independent evolutionary processes in different plant lineages. This result is consistent with the relevant findings in existing barley studies [31]. In addition, the Ka/Ks ratios were generally less than 1, suggesting that these homologous gene pairs underwent purifying selection during the evolutionary process, and their functions tend to be stable and conserved. Promoter cis-element analysis revealed that *SbCNGC* genes may respond to hormonal signals (e.g., ABA, JA, IAA) and abiotic stresses. Among them, functional elements such as ABRE, CGTCA element, MBS, LTR, etc., were widely distributed in the upstream promoter regions of several members, suggesting that they may play important regulatory roles in plants’ stress-sensing and response network. miRNA prediction further indicated that several miRNA families, such as miR395 and miR160, regulated the expression of SbCNGC genes through shear or translational repression, reflecting that their expression level was stable and conserved, suggesting that complex post-transcriptional regulatory mechanisms regulate their expression level. GO and KEGG enrichment analysis results further support the above findings, indicating that SbCNGC genes are significantly enriched in pathways related to voltage-dependent ion channel activity, potassium ion transport, electrical signal regulation, and stress responses. This aligns with existing literature on CNGC involvement in calcium signaling and stress responses, such as the close association of *AtCNGC2* and *AtCNGC4* genes in Arabidopsis with pathogen response and ion homeostasis regulation. *AtCNGC2* and *AtCNGC4* are closely linked to pathogen response and ion homeostasis regulation [32].

Previous studies have shown that *CNGC* genes are widely involved in plant responses to biotic and abiotic stresses; for example, *HvCNGC3* and *HvCNGC16* play a negative regulatory role in drought stress response. *TaCNGC14* and *TaCNGC16* mRNA accumulation is significantly affected by exogenous hormones such as abscisic acid (ABA), methyl jasmonate (MeJA), and salicylic acid (SA), suggesting a role in hormone signaling and/or sensing [17]. Arabidopsis *AtCNGC10* is involved in regulating salt tolerance in Arabidopsis [24]. Transcriptome analysis and real-time quantitative PCR revealed that the *SbCNGC1* gene exhibited sustained upregulation under both cold and heat stress, suggesting its potential key role in temperature-dependent Ca^2+^ signaling. Conversely, transient induction of genes such as *SbCNGC4*, *SbCNGC9*, and *SbCNGC22* during the early stages of stress suggests that plants may rely on rapid Ca^2+^/ion flux regulation mediated by these genes to maintain cellular homeostasis.

Calcium-dependent signaling pathways constitute one of the core regulatory networks in plant stress responses, particularly when confronting abiotic stresses. Extensive evidence indicates that plants utilize Ca^2+^ as a signaling molecule to regulate diverse physiological processes, thereby enhancing adaptability. For example, in *A. thaliana*, *CNGC20* plays an active role in regulating cold responses by promoting Ca^2+^ influx [33]; in rice, *OsCNGC14* and *OsCNGC16* enhance Ca^2+^ influx under cold or heat stress conditions [34]. Collectively, these studies indicate that CNGCs perform a central function in temperature-induced Ca^2+^ signaling pathways.

In this process, CNGC-type calcium channel proteins (e.g., *SbCNGC1*) may play a pivotal role. Recent years have seen further advances in understanding the relationship between Ca^2+^ signaling and stress responses. Quaratiello et al. [35] noted that melatonin exerts significant regulatory effects on plant oxidative stress responses, particularly under abiotic stress. Melatonin induces elevated Ca^2+^ concentrations within leaves, thereby modulating redox balance and enhancing plant stress tolerance. Its mechanism likely involves regulating reactive oxygen species (ROS) homeostasis and interacting with calcium signaling pathways to promote downstream stress-response gene expression. Based on this hypothesis, *SbCNGC1*, as a calcium channel protein, may participate in melatonin-mediated Ca^2+^ signaling regulation. By controlling Ca^2+^ flux within leaves, *SbCNGC1* may collaborate with melatonin to regulate ROS homeostasis and antioxidant enzyme gene expression, thereby enhancing plant adaptation to abiotic stresses.

Future studies could validate its function by generating *SbCNGC1* knockout or overexpression lines and subjecting them to various stress treatments, including temperature, salt, and drought. Additionally, integrated transcriptomic and metabolomic analyses could systematically elucidate the interactions between *SbCNGC1* and other signaling pathways, revealing a more comprehensive regulatory network.

## 5. Conclusions

This study analyzed the composition, structural characteristics, and evolutionary relationships of the sorghum CNGC gene family, while also investigating its expression patterns under various abiotic stresses. A total of 23 SbCNGC genes were identified, all possessing typical conserved domains and functional motifs, with uneven chromosomal distribution. Phylogenetic and structural analyses grouped them into five subfamilies (I-IV-b), with subfamily IV-b exhibiting species-specific expansion and strong structural conservation among the subfamilies. Integrating gene structure and promoter cis-element studies revealed a dynamic equilibrium between structural conservation and functional diversity within the SbCNGC family. Furthermore, miRNA target prediction and GO/KEGG enrichment analyses suggest SbCNGC genes may participate in diverse signaling and regulatory processes. Expression profiling further revealed tissue-specific expression patterns across different organs and temporal responses to low and high temperatures. Notably, SbCNGC1 exhibits sustained upregulation under both cold and heat stress, suggesting its potential as a key Ca^2+^ channel gene regulating multiple abiotic stress signaling pathways.

## Figures and Tables

**Table 1:** Basic information and subcellular localization of sorghum CNGC proteins.

Name	Gene ID	Chrom	Number of Amino Acid (aa)	Molecular Weight (Da)	Theoretical pI	Instability Index	Aliphatic Index	Grand Average of Hydropathicity	Subcellular Localization
SbCNGC1	XM_002466378.2	Chr1	729	81,533.04	9.34	57.47	94.92	0.102	Cell membrane
SbCNGC2	XM_002466707.2	Chr1	709	80,987.67	9.4	54.9	90.94	−0.121	Cell membrane
SbCNGC3	XM_021453318.1	Chr2	919	100,649.17	8.18	43.78	86.54	−0.141	Nucleus
SbCNGC4	XM_002460609.2	Chr2	713	82,328.6	9.19	49.21	91.67	−0.208	Cell membrane
SbCNGC5	XM_002458189.2	Chr3	885	99,321.75	7.04	39.84	94.42	−0.13	Nucleus
SbCNGC6	XM_021456300.1	Chr3	637	71,042.55	8.29	40.97	94.74	0.035	Cell membrane
SbCNGC7	XM_002456331.2	Chr3	530	61,024.6	8.64	46.83	93.62	−0.055	Cell membrane
SbCNGC8	XM_002458555.2	Chr3	677	75,481.41	9.69	53.39	90.96	−0.073	Cell membrane
SbCNGC9	XM_002453555.2	Chr4	729	83,534.61	6.3	38.1	88.55	−0.19	Cell membrane
SbCNGC10	XM_002453582.2	Chr4	642	73,383.79	8.4	51.93	94.95	0.008	Cell membrane
SbCNGC11	XM_021459169.1	Chr4	704	80,327.93	9.09	44.54	91.72	−0.079	Cell membrane
SbCNGC12	XM_002454023.2	Chr4	601	65,801.8	7.23	40.12	96.96	0.136	Cell membrane
SbCNGC13	XM_021458611.1	Chr4	697	80,905.02	9.69	55.42	90.98	−0.112	Cell membrane
SbCNGC14	XM_002454637.2	Chr4	701	80,045.61	9.08	41.34	92.54	−0.05	Cell membrane
SbCNGC15	XM_002447160.2	Chr6	721	82,522.16	9.15	47.07	86.16	−0.111	Cell membrane
SbCNGC16	XM_002442510.2	Chr8	749	85,154.09	9.54	49.73	84.15	−0.18	Cell membrane
SbCNGC17	XM_002441096.2	Chr9	838	92,793.4	7.09	34.04	95.76	−0.058	Nucleus
SbCNGC18	XM_021447292.1	Chr9	842	93,389.95	6.31	35.84	97.49	−0.031	Nucleus
SbCNGC19	XM_002441100.2	Chr9	837	93,301.97	6.06	32.3	96.43	−0.029	Nucleus
SbCNGC20	XM_021447943.1	Chr9	710	78,637.37	9.74	50.05	86.04	−0.1	Cell membrane
SbCNGC21	XM_002436576.2	Chr10	680	78,657.88	9.03	42.17	93.6	−0.125	Cell membrane
SbCNGC22	XM_021449564.1	Chr10	854	97,265.64	5.95	42.89	94.56	−0.15	Nucleus
SbCNGC23	XM_021449645.1	Chr10	691	80,003.45	9.47	51.91	89.77	−0.166	Cell membrane

## Data Availability

The RNA-seq data used in this study are available in the NCBI-SRA database (PRJEB22168 and PRJNA1159475). Additional data supporting the findings can be found in the Supplementary Materials.

## Supplementary Materials

The following supporting information can be downloaded at: https://www.mdpi.com/article/10.3390/genes16121405/s1, Table S1: RT-qPCR primer information; Table S2: Predicted secondary structure of SbCNGC protein; Table S3: Ka and ks Analysis; Table S4: Analysis of SbCNGC promoter homeopathic elements.
